# Autonomous Landmark Calibration Method for Indoor Localization

**DOI:** 10.3390/s17091952

**Published:** 2017-08-24

**Authors:** Jae-Hoon Kim, Byoung-Seop Kim

**Affiliations:** 1 Department of Industrial Engineering, Ajou University, Suwon 16499, Korea; 2 Infrastructure Protection Team, Korea Internet and Security Agency, Seoul 05717, Korea; kisainkbs@kisa.or.kr

**Keywords:** location-based services, data analytics, landmark, dead reckoning

## Abstract

Machine-generated data expansion is a global phenomenon in recent Internet services. The proliferation of mobile communication and smart devices has increased the utilization of machine-generated data significantly. One of the most promising applications of machine-generated data is the estimation of the location of smart devices. The motion sensors integrated into smart devices generate continuous data that can be used to estimate the location of pedestrians in an indoor environment. We focus on the estimation of the accurate location of smart devices by determining the landmarks appropriately for location error calibration. In the motion sensor-based location estimation, the proposed threshold control method determines valid landmarks in real time to avoid the accumulation of errors. A statistical method analyzes the acquired motion sensor data and proposes a valid landmark for every movement of the smart devices. Motion sensor data used in the testbed are collected from the actual measurements taken throughout a commercial building to demonstrate the practical usefulness of the proposed method.

## 1. Introduction

A location-based service (LBS) improves the economic and emotional utility of services in various user applications. LBSs are usually offered by cellular radio providers. In today’s LBSs, the typical cell-based location estimation takes advantage of various techniques: the global navigation satellite system (GNSS), Wi-Fi based fingerprint/triangulation, magnetic field fingerprints, and other complicated techniques for the sensitive measuring of the geographical location of a device. The LBS includes much useful information about specific locations in the user’s neighborhood. Maps and navigation are the essential service capabilities that permit the fundamental location management. Map and navigation services have sufficient expandability to provide asset tracking, autonomous driving and context aware services. Augmented reality (AR) is a prospective LBS service. An accurate geographical location enhances AR applications. More geographically appropriate information or context can be added to the display of a mobile device. The additional context, which is highly related to locations, provides vast opportunities in gaming, advertising, and social applications. The strong usability offered by the LBSs provides huge market expansion. In addition to popular map/navigation services, food delivery, car sharing, and many other Internet of Thing applications are prevailing among the masses. However, the popularity of LBS is generally focused on the outdoor environment. Indoor spaces require high location precision because the environmental contexts change with a much finer spatial granularity. Just a five-meter difference may indicate just two different aisles in a general grocery store. Many research trials have been implemented to achieve affordable location accuracy. Image-based location tracking is a promising approach. Guoyu et al. [1,2] proved that the high location accuracy can be achieved by using captured video for indoor environments. Hongbo et al. [3,4] developed an advanced Wi-Fi fingerprint location estimation method using peer assistance. Moreover, Yuanchao et al. [5] enhanced the Wi-Fi fingerprint method by adopting a gradient for each Wi-Fi fingerprint to eliminate biased measurements. Probabilistic fingerprint location estimation methods are also proposed. The studies of Seco et al. [6], Roos et al. [7], and Youssef et al. [8] estimated the probability distribution of the measured fingerprints given the user locations. Coverage estimation models were developed by Muller et al. [9] and Koski et al. [10]. They estimated the probabilistic coverage of Wi-Fi access points and then picked the user locations using probabilistic intersections of access point coverages. As recent approaches, machine learning algorithms were applied to enhance fingerprint location estimation. Feng et al. [11] suggested a support vector machine model and an interpolation method to reduce estimation errors. Sanchez-Rodriguez et al. [12] proposed a multiple weighted decision tree model for indoor location estimation.

The fingerprint location estimation is not restricted to Wi-Fi signals. Magnetic field fingerprints use magnetic field distributions inside buildings. The metal structure of the indoor environment cause magnetic field disturbances [13,14]. The results of previous studies have provided relatively stable location estimation methods [15,16,17]. The study of Torres-Sospedra et al. [18] presented stable continuous estimation using small mobile devices. However, Gozick et al. [15] showed the the magnetic field fingerprint restrictions caused by limited measuring features: the direction of the magnetic field is restricted in two dimensions in many real life scenarios. Another challenge if using a magnetic field for location estimation with mobile devices is significant variability of the magnetic field [17]. Recent studies have tried to address these challenges. Wang et al. [19] applied a weighted particle filter to reduce the irregular fluctuation of magnetic fields. Madson and Rahanani [20] suggested an adaptive dipole model for error compensation.

Sensor-based location estimation is another general approach for indoor spaces. An inertial measurement unit (IMU) that is embodied in every smart device measures the velocity, orientation, and acceleration of the device. Using the IMU, we can estimate the movement of objects. This estimation method is named dead reckoning [21,22,23,24,25]. By using the pedestrian working model, we can estimate the movement or orientation of a device without any infrastructure. Fuchs et al. identified the common requirements of indoor tracking in mission critical scenarios and introduced the basic techniques for IMU location estimation [26]. Beauregard et al. proposed a combined approach of pedestrian dead reckoning and GPS location estimation [27]. An accelerometer provides signals and a neural network predicts the step length for relative indoor positioning. Jiménez et al. used a low-performance micro-electro-mechanical sensor (MEMS) attached to the foot of a person [28]. This sensor has a triaxial accelerometer, a gyroscope, and a magnetometer. They implemented and compared most relevant techniques for user movement such as step detection, stride estimation, etc.

**Figure 1:**
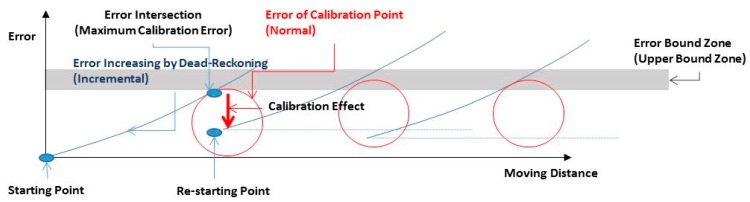
Calibration effect.

A single use of the IMU-based dead reckoning has a characteristic error generation pattern—accumulation of error. The dead-reckoned tracking is accurate in the beginning, however, the errors from the truth accumulate over time because of the noise in the mobile sensors. Periodic error calibrations are necessary to maintain the quality of estimation. Figure 1 illustrates the error generation characteristics of an IMU after appropriate calibration.

The error generation pattern of the IMU-based location estimation is simply incremental. The blue line in Figure 1 shows this incremental error pattern. We can restrict the increment in error accumulation within a certain limit by calibrating the IMU. The red circle in Figure 1 shows the natural error of a calibration method. By using an appropriate calibration method, we can achieve an acceptable limit of the estimation quality.

A popular calibration method uses a Wi-Fi system [29,30]. Affordable precision is attainable using a pervasive Wi-Fi system with meticulous signal calibration. However, Wi-Fi signal patterns are usually unstable. The unstable pattern comes at a high cost caused by frequent re-establishment of calibration points. This tradeoff between the calibration overhead and the accuracy has been an important challenge to deploying Wi-Fi calibration for indoor location estimation. If one could identify other appropriate means of calibration, the IMU-based dead reckoning could be sufficiently applicable, even in indoor environments.

Activity recognition and environment sensing demonstrate the ability to recognize the user behavior and spatial significance. For instance, the IMU can detect user activities such as walking, turning a corner, and stepping up and down stairs, whereas magnetometers can detect significant magnetic signals. If these sensory signatures are used for context awareness, they can be referred to as location estimation as well. These signatures can be used as landmarks to calibrate IMU-based dead reckoning [31]. The essential point of localizing the signatures (i.e., finding landmarks) is the more accurate detection of sensory signatures that have concrete uniqueness around the neighboring spatial field. To be a landmark the sensory signature of a location should be statistically differentiated. In this article, we propose a structural design of indoor spaces and a statistical procedure to identify significant landmarks for indoor dead reckoning. First, we analyze and understand the extensive sensory signatures of natural landmarks. Then, we propose an autonomous procedure for landmark identification. The autonomous procedure is especially reinforced by an arbitrary but large number of mobile devices. They identify the landmarks repeatedly, which can enhance the quality of landmark identification. The proposed statistical process of autonomous procedure improves the localization accuracy over time.

## 2. Materials and Methods

### 2.1. Structural Landmark Identification

**Figure 2:**
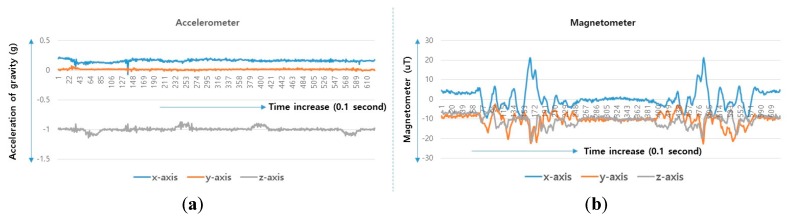
Accelerator/magnetometer fluctuation pattern corresponding to elevator: (**a**) Accelerometer, (**b**) magnetometer.

A user performs a predictable motion when passing through a specific structure, such as a flight of stairs, elevator, entrance, or escalator, in an indoor space. The predictable motion can be converted to a sensory signature by mobile devices: a multi-dimensional combination of sensory data postulates that signatures are likely to emerge for specific structures. For instance, the fluctuations in the gyroscope and magnetometer measurements are mapped to their corresponding physical locations such as an escalator. These spatial sensory signatures determine the “structural” landmarks. To recognize an elevator landmark, we can monitor the fluctuation variance on every axis of the magnetometer. The specific magnetometer fluctuations are observed for every stop over an elevator movement (see Figure 2).

Similar to an elevator landmark, we can categorize other types of typical structural landmarks: corridors, corners, stairs, and escalators. Table 1 lists the observation and visualization of the measured sensory signatures for each structural landmark.

To confirm each sensory signature as a valid structural landmark, we propose a validity test using the landmark confirming criteria. The landmark confirming criteria listed in Table 2 are obtained by conducting extensive empirical tests in indoor spaces. We develop a custom sensory signature collecting application for three types of mobile devices: a Samsung Galaxy S7 (Samsung Electronics, Seoul, Korea), a Sony Xperia XZ (Sony Corporation, Tokyo, Japan), and an LG G5 (LG Electronics, Seoul, Korea). The raw sensor data measured by the embodied IMU and magnetometer are transformed into sensory signature values for each measurement time interval (usually 100 ms). The gathered sensory signatures are transferred to a Microsoft Excel program for visualization after the whole signature gathering process. The tested indoor space is the second floor of a college building that includes 15 corridors, 10 corners, three sets of stairs, two escalators, and three elevators. We collect sensory signatures at least 20 times for each structure. An experimenter holds mobile devices with a handheld pose (generally, when a person looks for a way using navigation application in an indoor space, he or she holds a mobile device in hands and looks at the screen with his or her eyes).

By using the statistical analytics (i.e., one-way ANOVA), we define the key criteria to identify the valid structural landmarks. The key criteria (e.g., *z*-axis accelerometer for corridors or stairs) are identified within a significance level of 5%. If an observed average sensory signature for the key criteria is within the confidence interval (i.e., $\mathrm{average}\pm z_{0.025}\times \sqrt{\mathrm{variance}}$, $z_{0.025}=1.96$), the observed signature can be considered a structural landmark under a statistical confidence of 95%.

Structural landmarks provide effective calibration points for indoor location estimation. However, the number of structural landmarks is often not sufficient to sustain the desirable accuracy of location estimation. The increasing density of landmarks will reduce the location estimation error by frequent calibration. Thus, we focus on another type of landmark—spontaneous landmarks.

### 2.2. Spontaneous Landmark Identification

Indoor spaces offer ambient sensory signatures across multiple sensors. For instance, the metallic objects in a certain location may generate unique and reproducible patterns on a magnetometer. These ambient sensory signatures generally may not be recognized a priori. A different indoor space will have a different distribution of signatures. Although there is no theoretical proof, empirical observations indicate that an indoor space has sufficient ambient signatures from sound, light, magnetic field, temperature, and radio signals. An effective method to identify the ambient signatures is definitely useful for frequent calibrations. The identified ambient signatures can be used to determine the “spontaneous” landmarks that supplement the limited amount of structural landmarks.

Recognizing distinct sensory signatures is essential for discovering spontaneous landmarks. Sensory signatures are characterized by the mean, max, min, and variance similar to structural landmarks. Our objective is to identify distinct signatures that have less similarity to the signatures of neighboring locations. Distinct signatures are generally identified using a sensory signature gathering process. A sensory signature gathering application installed in smart devices collects sensory signatures continuously according to the walking path of a pedestrian. A similarity test is applied to two time-consecutively collected sensory signatures: one signature at time interval t and the other at *t* − 1. If the correlation between the two consecutive signatures is greater than a similarity threshold, we consider the signature at time interval *t* as statistically distinct, and the location of the distinct signature is considered as the candidate for a spontaneous landmark.

The sensory signatures measured at time interval *t* are described using a vector form that has three tuples. The first tuple comprises the signatures of an accelerometer as expressed by (1):

(1) $$f_{accelerometer}^{t}=(a_{x-mean}^{t},a_{x-\min}^{t},a_{x-\max}^{t},a_{x-\mathrm{vari}ance}^{t},a_{y-mean}^{t},a_{x-\min}^{t},a_{y-\max}^{t},a_{y-\mathrm{var}iance}^{t},a_{z-mean}^{t},a_{z-\min}^{t},a_{z-\max}^{t},a_{z-\mathrm{var}iance}^{t})$$

where $a_{x-mean}^{t}$ denotes the mean value of the x-axis accelerometer at time interval *t* and the other three elements of the *x*-axis accelerometer denote the min, max, and variance. Similarly, the elements for *y*-axis and *z*-axis accelerometers are contained in the first tuple, $f_{accelerometer}^{t}$. The tuples for gyroscope and magnetometer are defined as shown in (2) and (3):

(2) $$f_{gryoscope}^{t}=(g_{x-mean}^{t},g_{x-\min}^{t},g_{x-\max}^{t},g_{x-\mathrm{var}iance}^{t},g_{y-mean}^{t},g_{x-\min}^{t},g_{y-\max}^{t},g_{y-\mathrm{var}iance}^{t},g_{z-mean}^{t},g_{z-\min}^{t},g_{z-\max}^{t},g_{z-\mathrm{var}iance}^{t})$$

(3) $$f_{magnetometer}^{t}=(m_{x-mean}^{t},m_{x-\min}^{t},m_{x-\max}^{t},m_{x-\mathrm{var}iance}^{t},m_{y-mean}^{t},m_{x-\min}^{t},m_{y-\max}^{t},m_{y-\mathrm{var}iance}^{t},m_{z-mean}^{t},m_{z-\min}^{t},m_{z-\max}^{t},m_{z-\mathrm{var}iance}^{t})$$

A typical sensory signature is a combination of three tuples: $s^{t}$ = {$f_{accelerometer}^{t}$, $f_{gryoscope}^{t}$, $f_{magnetometer}^{t}$}. The proposed similarity test is conducted with two sensory signatures at consecutive time intervals. Each time interval holds separated portion on the time axis. The two consecutive time intervals are not overlapped. The square of the Euclidean distance (i.e., $d^{2}(s^{t-1},s^{t})$ given by (4)) of the two time-consecutive signatures ($s^{t-1}$, $s^{t}$) presents the statistical difference of the two sensory signatures:

(4) $$d^{2}(s^{t-1},s^{t})={\left(s^{t-1}-s^{t}\right)}^{2}$$

Each element of a sensory signature has a normally distributed measurement error. The central limit theorem [32] guarantees the normal distribution of relatively large number (usually, more than 20) of independent sensor measurements. Thus, each element of sensory signature follows a normal distribution. Then, the vector difference of two signatures ($s^{t-1}-s^{t}$) also follows a normal distribution. Each element of vector $s^{t-1}-s^{t}$ follows a normal distribution. Then, $d^{2}(s^{t-1},s^{t})$, the square summation of elements of vector $s^{t-1}-s^{t}$ follows chi-squared distribution [33] with degrees of freedom m, where m is the number of independent elements of a vector (i.e., $d^{2}(s^{t-1},s^{t})\sim \chi^{2}(m)$, mean of $\chi^{2}(m)$ is m and variance is 2m). The chi-square distribution with m degrees of freedom is the distribution of a sum of the squares of *m* independent normal random variables. By the statistical finding of Fisher [34], $\sqrt{2d^{2}(s^{t-1},s^{t})}$ follows approximate normal distribution with mean $\sqrt{2m-1}$ and a unit variance. The significant difference between two sensory signatures can be determined by its statistical characteristics. When a value of $\sqrt{2d^{2}(s^{t-1},s^{t})}$ is over the given threshold, the two sensory signatures, $s^{t-1}$ and $s^{t}$, are considered significantly different. Note that, the time interval is usually 100 ms. Because of the highly sensing frequency of embodied IMU and magnetometer (100 Hz–400 Hz for usual mobile devices), 100 ms time interval provides 10–40 sensing opportunities for single time interval. It is the sufficient number to obtain valid sensory signatures for every time intervals.

**Figure 3:**
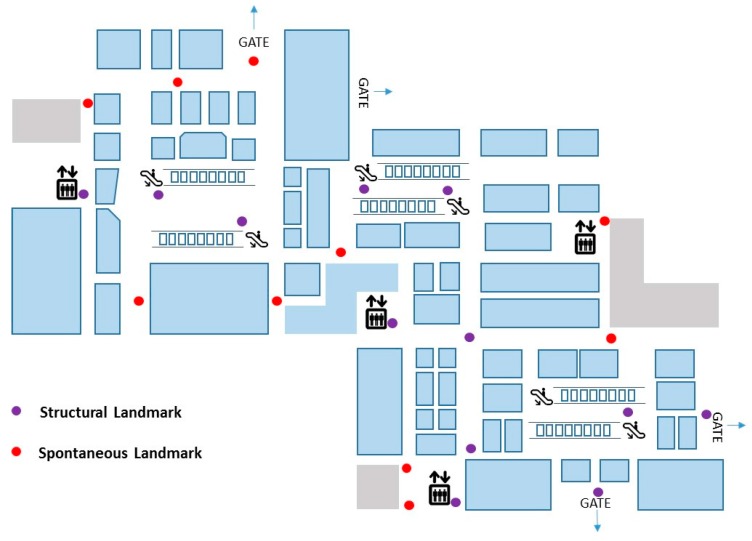
Example of landmarks detection.

For obtaining the spontaneous landmarks, we subject the sensory signatures to the continuous process of a similarity test. Once the similarity test has approved, each of the resulting distinct sensory signatures is expected to contain a unique location. We detect the unique sensory signatures and record their ground truths to localize the spontaneous landmarks. We build an entire map of landmarks by the combining both the structural and spontaneous landmarks. Figure 3 shows an example of landmarks. The illustrated floor plan is obtained from a commercial departmental store in Incheon, Korea. We detected 11 structural landmarks and 10 spontaneous ones.

**Figure 4:**
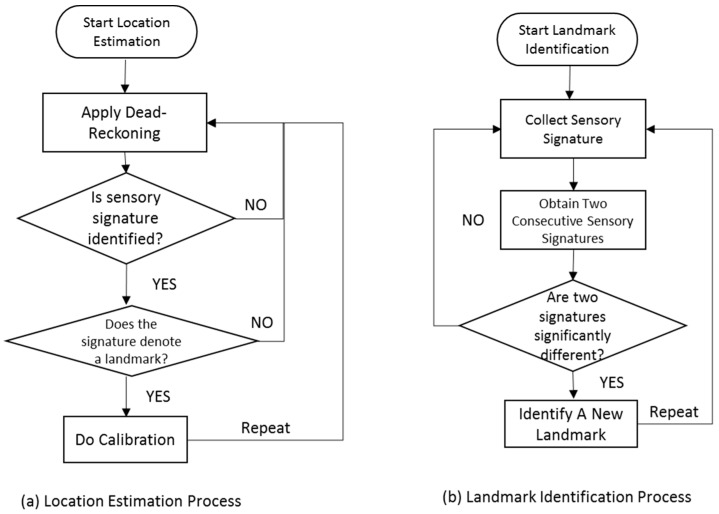
Process of landmark identification and location estimation.

The ground truths that are used to localize the spontaneous landmarks are collected by voluntary activities. A simple sensory signature gathering application generates an alarm whenever distinct sensory signatures are detected. A user can record the geographical ground truths for the detected signatures by pinpointing over the floor plan. In addition, the ground truth can be estimated by the autonomous actions of a sensory signature gathering application. The sensory signature gathering application automatically records the current location obtained by dead reckoning for a detected signature. The autonomous recording has a relatively large error compared to voluntary pinpointing. For the estimation of a location by dead reckoning, repetitive collection and averaging can reduce the error of the ground truth estimation. Figure 4 illustrates the entire process of landmark identification and location estimation. The identified landmarks are continuously used for recalibration. Periodic recalibration can achieve an acceptable upper bound of location errors.

**Figure 5:**
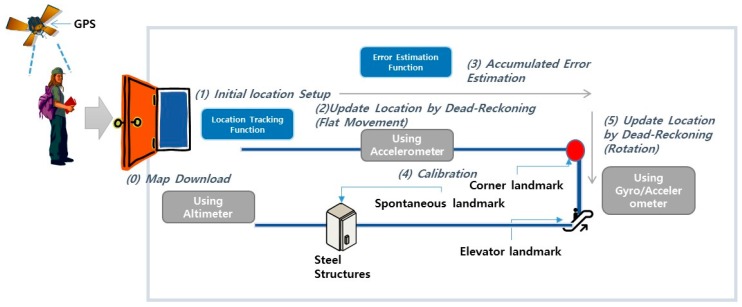
User location estimation by continuous calibration.

The floor plan with the entire landmark information is downloaded when a user reaches an indoor space. We can dead reckon our indoor locations with frequent recalibration (see Figure 5). The recalibration is activated by both the structural and spontaneous landmarks. 

## 3. Results

**Figure 6:**
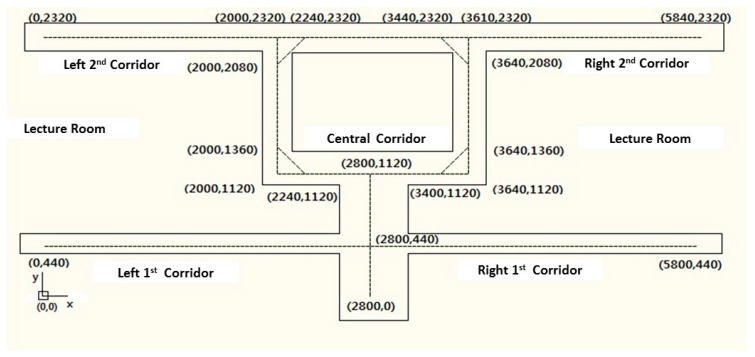
Test floor of small area.

To demonstrate the practical usability of the proposed landmark calibration method, we identified the structural and spontaneous landmarks for two different types of buildings. A large area cannot be appropriate to test and enhance the applicability of the landmark identifying process. Thus, we first apply the developed similarity test in a relatively small area. The test area is the second floor of a college building. Figure 6 illustrates the details of the floor. The bottom-left point is the origin of the coordinates. The distances from the origin are represented in centimeters. To identify the landmarks, we repeatedly wander around the test floor. After the determination of landmarks, we measure the location estimation error for total seven paths. Each path has ten location measuring points.

**Figure 7:**
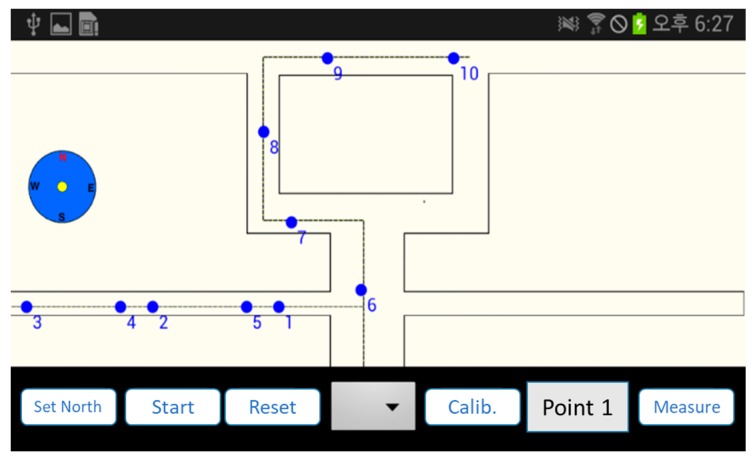
Test tool.

The similarity test processes on two time-consecutive sensory signatures (one signature is measured at time t and the other is measured at t−1). The difference of two signatures (i.e., $\sqrt{2d^{2}(s^{t-1},s^{t})}$ in Section 2.2) is normally distributed with mean $\sqrt{2m-1}$ and unit variance (i.e., σ=1). Then, we apply $\sqrt{2m-1}+k$ as a useful threshold value. The constant value, k, determines the strictness of the similarity test. Suppose that k is equal to 1, the two sensory signatures are determined to be statistically different only when they have a difference belonging to the upper 15.8% of the difference distribution. In the cases of k=1.5 and k=2, we consider the upper 6.7% and 2.2% of the total difference distribution as the similarity threshold, respectively. In this experiment, we indicate the results corresponding to k= 1, 1.5, and 2 to demonstrate the usefulness of the similarity test. A mobile application tool is developed for the test. The application obtains the landmarks, sets up a test path, and performs dead reckoning with calibration as shown in Figure 7.

**Figure 8:**
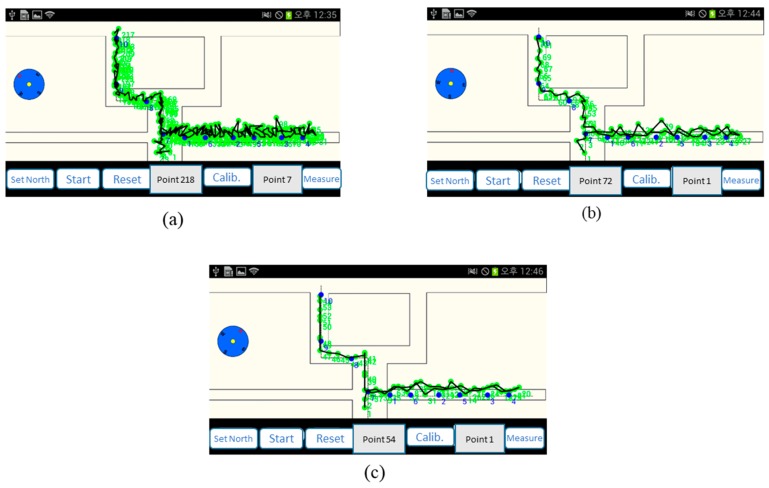
Traces for *k* value: (**a**) k=1, (**b**) k=1.5, (**c**) k=2.

Figure 8 illustrates the difference corresponding to different *k* values: k=1 has the largest number of spontaneous landmarks and k=2 has the least number of spontaneous landmarks. The actual footprints of users are illustrated using the green dots. The black lines show the average traces for multiple experiments per single path.

The location errors for each *k* value are listed in Table 3. The number of spontaneous landmarks increases with a decrease in *k* values, whereas the number of structural landmarks is constant. The location estimation errors are not minimized when the number of landmarks is the largest. However, the location errors are minimized at the median of *k*.

**Figure 9:**
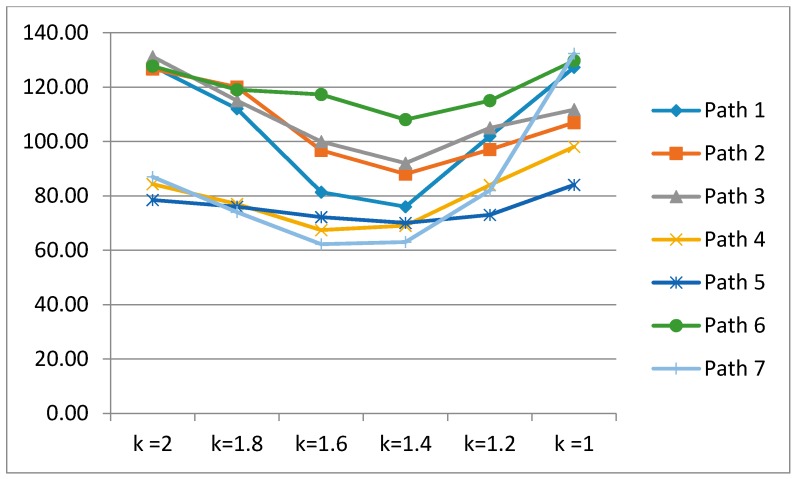
Error charts for *k* values (unit of *y*-axis: cm).

Figure 9 illustrates the patterns of location errors for each experimental path. Location errors are minimized at the median of *k*. We expand the k value into {1, 1.2, 1.4, 1.6, 1.8, 2} to enhance the visibility of the trends. If k= 1.2, we consider the upper 11.5% of the total difference distribution as the similarity threshold. Similarly, upper 8.1% when k= 1.4, 5.5% when k= 1.6, and 3.6% when k= 1.8 are considered to similarity thresholds.

Next, we extended the landmark calibration method to a commercial department store located in Incheon, Korea. The floor area of the target floor is 9428 m^2^. Three escalators and four elevators are included in the target floor. In addition, the corners are included over the seventy places in the target floor (see Figure 3).

We listed the results of three representative similarity thresholds: k= 1, 1.5, and 2. Table 4 lists the number of structural and spontaneous landmarks, recalibration, and errors of location estimation for ten paths.

**Figure 10:**
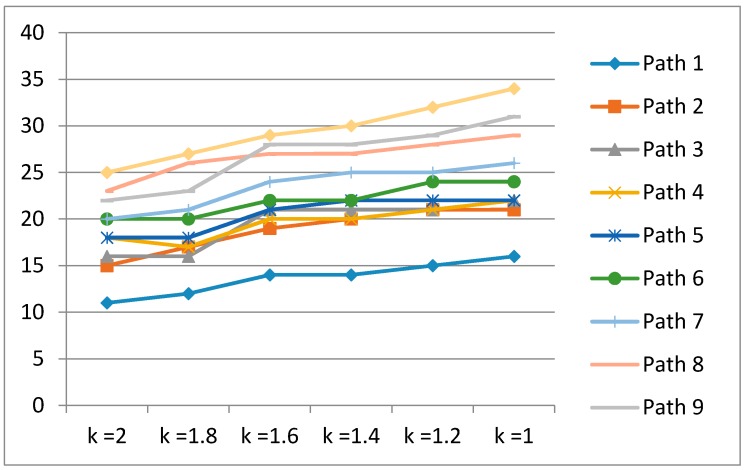
Number of recalibrations for *k* values in large test area (unit of y-axis: recalibration frequency).

Figure 10 illustrates the number of recalibrations for each experimental path. The number of recalibrations increases with decreasing *k* values.

**Figure 11:**
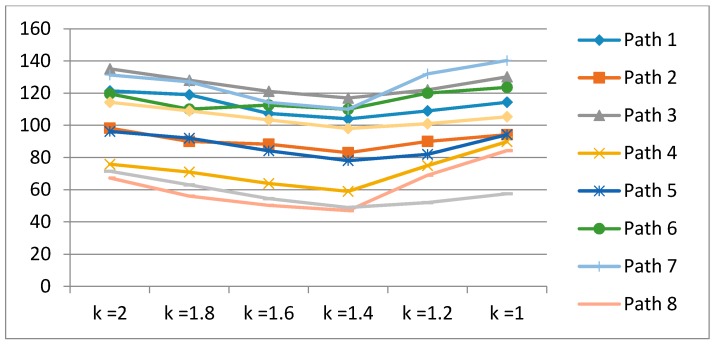
Error charts for *k* values in large test area (unit of *y*-axis: cm).

However, frequent recalibration does not guarantee the quality of location estimation. The location errors are minimized under the median of recalibrations (see Figure 11). We also expand the k value into {1, 1.2, 1.4, 1.6, 1.8, 2} to indicate a more explicit trend of recalibration.

**Figure 12:**
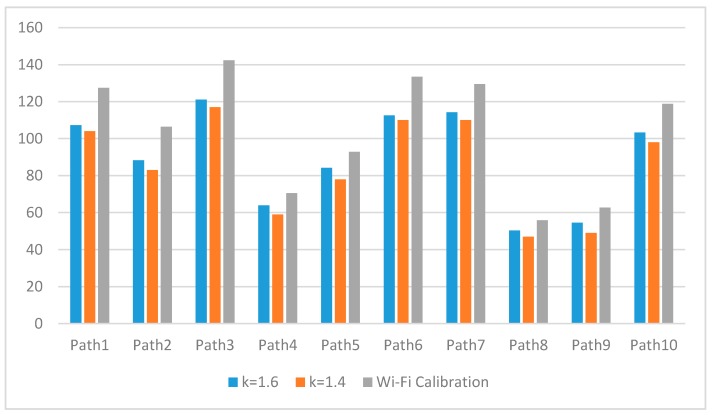
Comparison with Wi-Fi calibration (unit of *y*-axis: cm).

Figure 11 illustrates the patterns of location errors for each experimental path. The median of *k* minimizes the location errors. The results of the calibration method using a Wi-Fi system [29,30] are illustrated in Figure 12 to show the excellence of the proposed method. The number of Wi-Fi recalibrations are slightly different for each test path: the maximum number of recalibration is 27 times for path 10 and the minimum is 11 times for path 1. Figure 12 shows the performance comparisons of Wi-Fi calibration and the proposed autonomous landmark calibration (*k* = 1.4 and *k* = 1.6). The proposed method illustrates the superior results for average 17.8% enhancement.

To concretely determine a landmark in the extended department store experiment, we apply collective intelligence by voluntary activities. Repeated detections of same signature on the same location is needed to be a landmark. In addition, when we detect a landmark signature during navigation, we check the stored ground truth of the landmark. If the ground truth of the detected landmark is far from the current location of the devices, we determine the detected landmark as an improper one. Then, we inactivate the calibration process.

## 4. Discussion

The proliferation of mobile communication and smart devices has drawn significant attention to Location-Based Services. Accurate location estimation is one of the most essential components for the usability of LBSs. Especially in an indoor environment, the traditional location estimation methods such as pure dead reckoning have the limitation of error accumulation over time. Moreover, solution providers do not share significant technical advances publicly. The dead reckoning with frequent recalibration is a beneficial technique for expanding the LBS capability. The landmark points, structural or spontaneous, activate recalibration. The corner points, stairs, escalators, and elevators are representative structural landmarks. The usefulness of structural landmarks can stimulate the expansion of spontaneous landmarks. A statistical similarity test identifies various spontaneous landmarks. The sensory signatures of geographical locations are represented using a vector form. The difference between the two sensory signature vectors can be evaluated by conducting a similarity test. This autonomous landmark finding procedure is used to reduce cost while keeping a affordable estimation quality. The theoretical achievement of statistical analysis strengthens the advantage of LBS application especially for indoor location estimation. In addition, the proposed landmark calibration can be tightly coupled to traditional Wi-Fi calibration. The well-trained Wi-Fi calibration points can be added as important landmarks in our proposed method. All the sensory signatures were acquired through the actual measurements, which validates the practicality of the proposed method.

## Figures and Tables

**Table 1:** Determinants and visualization of sensory signature.

Structural Landmark	Observation	Visualization
**Corridor**	Typical vibration/fluctuation on the *z*-axis of the accelerometer	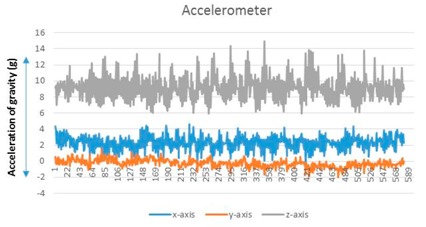
**Corner**	Changes corresponding to a corner are measured by the yaw-axis (*z*-axis) of the gyroscope over the ± 1.5 rad/s	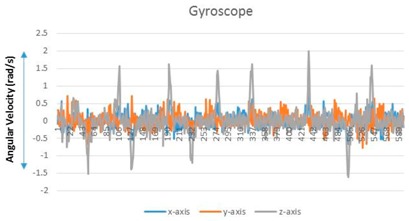
**Stair**	A large fluctuation variance in the *z*-axis of the accelerometer (approximately greater than twice the fluctuation variance corresponding to a corridor)	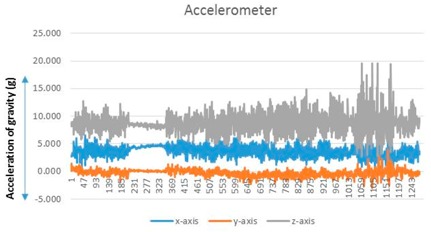
	The change in the direction measured by the yaw-axis of the gyroscope (Turning into a corner is observed additionally)	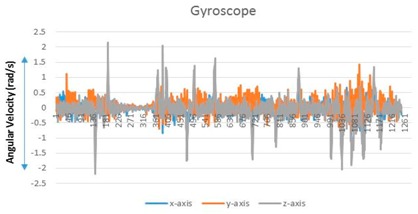
**Escalator**	The on–off pattern of the fluctuation variance on the *z*-axis of the accelerometer over a relatively long duration (less than 20 s)	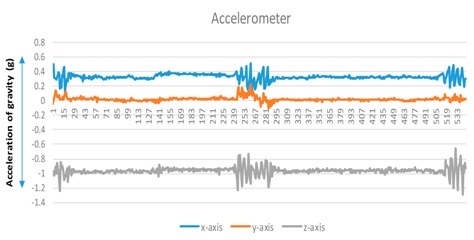

**Table 2:** Central values for landmark confirming criteria.

Signature	Key Criteria	Mean	Maximum	Minimum	Variance
Stationary	*z*-axis accelerometer (m/s^2^)	8.973	9.32	8.707	0.009
Corridor	*z*-axis accelerometer	9.1099	14.9578	5.9023	2.3549
Corner	*z*-axis gyroscope (rad/s)	0.0151	2.0107	−1.603	0.1741
Stair	*z*-axis accelerometer	8.7039	19.6085	2.6965	4.0408
Escalator	*z*-axis gyroscope	0.0298	3.0201	−2.703	0.2132
Elevator	*z*-axis magnetometer (µT)	−10.5621	−5.1028	−21.8373	7.721

*Notes*: (1) A sensory signature for stationary state is included for comparison. (2) The *z*-axis accelerometer value is generally less than 1 G (9.8 m/s^2^) because an experimenter holds mobile devices with a slightly tilted handheld pose. (3) The significant change of gyroscope value is caused by a turning movement. To prevent confusion caused by changing direction (left turn or right turn), we use absolute gyroscope measurement values.

**Table 3:** Error distribution according to landmark density (unit: cm).

	*k* = 2			*k* = 1.5			*k* = 1		
	Error	Number of Landmarks		Error	Number of Landmarks		Error	Number of Landmarks	
		Structural	Spontaneous		Structural	Spontaneous		Structural	Spontaneous
**Path1**	127.69	3	5	80.33	3	7	127.24	3	10
**Path2**	126.66	3	4	94.71	3	7	106.93	3	9
**Path3**	131.16	4	6	97.94	4	7	111.71	4	9
**Path4**	84.29	4	7	64.38	4	8	98.05	4	11
**Path5**	78.47	5	7	71.15	5	9	84.04	5	13
**Path6**	127.69	5	8	116.28	5	10	129.73	5	14
**Path7**	86.95	7	10	59.17	7	13	132.32	7	16

**Table 4:** Error distribution according to recalibrations for large area (unit: cm).

	*k* = 2		*k* = 1.5		*k* = 1	
	Error	Number of Recalibration	Error	Number of Recalibration	Error	Number of Recalibration
**Path1**	121.31	11	106.31	14	114.31	16
**Path2**	98.24	15	86.24	19	94.24	21
**Path3**	135.12	16	119.12	21	130.12	22
**Path4**	75.87	18	62.87	20	89.87	22
**Path5**	96.19	18	82.19	21	94.19	22
**Path6**	119.56	20	102.56	22	118.56	24
**Path7**	131.29	20	112.29	24	140.29	26
**Path8**	67.31	23	48.31	27	84.31	29
**Path9**	71.49	22	53.49	28	57.49	31
**Path10**	114.35	25	102.35	29	105.35	34
